# Review of the Occurrence of Anti-infectives in Contaminated Wastewaters and Natural and Drinking Waters

**DOI:** 10.1289/ehp.11776

**Published:** 2009-01-22

**Authors:** Pedro A. Segura, Matthieu François, Christian Gagnon, Sébastien Sauvé

**Affiliations:** 1 Département de Chimie, Université de Montréal, Montréal, Québec, Canada;; 2 Aquatic Ecosystem Protection Research Division, Environment Canada, Montréal, Québec, Canada

**Keywords:** antibacterials, antibiotics, antimicrobials, aquatic environment, drinking water, groundwater, resistance, surface water, wastewater

## Abstract

**Objective:**

Anti-infectives are constantly discharged at trace levels in natural waters near urban centers and agricultural areas. They represent a cause for concern because of their potential contribution to the spread of anti-infective resistance in bacteria and other effects on aquatic biota. We compiled data on the occurrence of anti-infectives published in the last 24 years in environmental water matrices. The collected information was then compared with the available ecotoxicologic values to evaluate potential environmental concerns.

**Data sources:**

We used Web of Science and Google Scholar to search for articles published in peer-reviewed journals written in the English language since 1984.

**Data extraction:**

Information on compound concentrations in wastewaters and natural and drinking waters, the source of contamination, country of provenance of the samples, year of publication, limits of quantification, and method of analysis was extracted.

**Data synthesis:**

From the 126 different substances analyzed in environmental waters, 68 different parent compounds and 10 degradation products or metabolites have been quantified to date. Environmental concentrations vary from about 10^−1^ to 10^9^ ng/L, depending on the compound, the matrix, and the source of contamination.

**Conclusions:**

Detrimental effects of anti-infectives on aquatic microbiota are possible with the constant exposure of sensitive species. Indirect impact on human health cannot be ruled out when considering the potential contribution of high anti-infective concentrations to the spreading of anti-infective resistance in bacteria.

Anti-infectives are substances that in small doses can inhibit the growth or the survival of microorganisms without affecting the host (Foye et al. 1995; Korolkovas 1976). They comprise several classes of biologically active compounds of natural or seminatural origin such as antibiotics (e.g., β-lactams, tetracyclines) or from synthetic sources such as antimicrobials (e.g., sulfonamides, quinolones) and some antifungals (e.g., azoles). These substances are used to treat infections or to prevent them in humans, animals, and food-producing insects and plants. In some food animals, subtherapeutic amounts of anti-infectives are also used as feed additives to reduce their susceptibility to stress-related diseases and to enhance growth (Kumar et al. 2005). Anti-infectives have been recognized as the most significant therapeutic breakthrough in the history of medicine (Levy 1992), and estimates indicate that between 100 and 200 × 10^6^ kg of these compounds are consumed annually worldwide (Wise 2002).

The first report on the appearance of anti-infectives in the environment was published in 1983 (Watts et al. 1983). Further studies were limited by the availability of sufficiently sensitive techniques, and it was not until the late 1990s and early 2000 that sensitive methods of trace analysis were first introduced (Golet et al. 2001; Hirsch et al. 1998; Lindsey et al. 2001). The occurrence of these compounds in the environment has raised concern about their potential role in the dissemination of anti-infective resistance in bacteria and the impact of their biological activity on the health of aquatic ecosystems (Daughton and Ternes 1999).

To date, no comprehensive review on the occurrence of anti-infectives in the environment has been published. Therefore, the main goal of this work is 3-fold: *a*) to summarize published information on the environmental concentrations of anti-infectives in water matrices (wastewaters and natural and drinking waters) in the last 24 years; *b*) to summarize the present body of knowledge on the presence of anti-infectives in the aquatic environment; and *c*) to estimate possible effects of anti-infectives in the environment by comparing environmental concentrations with environmental effective concentrations.

Here we do not discuss fate and occurrence in soils or sediments, as other papers have already commented on this subject (Thiele-Bruhn 2003; Tolls 2001). Analytical methods (Díaz-Cruz and Barceló 2005; Fatta et al. 2007; Ternes 2001), the fate in waste-water treatment plants (Jones et al. 2005; Petrović et al. 2003), and ecotoxicity (Crane et al. 2006; Jjemba 2006; Jones et al. 2004) are also among the subjects that will not be discussed in detail, given the excellent earlier published reviews.

## Sources and transport

Several sources of anti-infectives in the environment have been identified, such as manufacturing wastes (Babić et al. 2007; Larsson et al. 2007), improper disposal of unused medication (Bound and Voulvoulis 2005), and landfill leachates (Holm et al. 1995). However, it has been recognized that the excretions of people or animals under treatment are the foremost source of anti-infectives in the aquatic environment (Hirsch et al. 1999). Hence, anti-infectives reach the aquatic environment through two main routes: urban and agricultural.

In the urban route, the anti-infectives excreted [for some compounds, as much as 90% in the parent form (Jjemba 2006)], washed off (in the case of topical formulations), or discarded by people in households, hospitals, or industries will end up in sewage. Once in wastewater, anti-infectives are discharged directly to surface waters or transported by sewers to wastewater treatment plants (WWTPs). During this process, the anti-infective loads in sewage may be diluted by the mixing with used water containing none of these substances (Alexy 2004). Anti-infectives may also reach the aquatic environment directly because of leaking sewers and sewer overflows (Sedlak et al. 2004). Compounds arriving at WWTPs may be eliminated from wastewater, depending mainly on their capacity to associate with particulate matter (which influences their removal by physicochemical or biological treatments) and their susceptibility to biological transformation (which certainly affects their elimination by biological treatment) (Ternes and Joss 2006). Partial biodegradation and mineralization of anti-infectives in WWTPs is possible, as bacteria may cometabolize these substances or use them as a source of carbon and energy to grow (Ternes et al. 2004). Substances having a lower affinity for solids and higher resistance to biotransformation will be subsequently discharged into streams (Roberts and Thomas 2006). Substances sorbed to sludge during treatment in WWTPs can also reach the environment by the application of sewage sludge in agricultural fields or by leaching in land-fills. For these reasons, WWTPs are the main entry point of urban anti-infectives into the aquatic environment (Glassmeyer et al. 2008; Ternes et al. 2004)

In the agricultural route, anti-infectives present in animal excreta may reach the aquatic environment by drainage and runoff to surface water and by percolation to ground-water. Studies have shown that compounds may be transported by the aqueous phase or bound to particulates in suspension (Kay et al. 2004, 2005), and this pathway is enhanced mainly because of land application of manure (Alexy 2004; Kumar et al. 2005). Substances retained and progressively accumulated in soils can be gradually released into the aqueous phase; agricultural soils may therefore act as environmental reservoirs for anti-infectives (Lee et al. 2007; Rooklidge 2004). These substances can also reach natural waters directly by leaking from manure storage structures or constructed lagoons (Meyer 2004) or through dust (Hamscher et al. 2003). Compounds used in aquaculture are often released directly into surface waters by leaching from food pellets, fish feces, or pond sediments (Cabello 2006; Lee et al. 2007). Anti-infectives sprayed on fruit plants may reach the aquatic environment; however, this pathway has not yet been documented. Therefore, agricultural activities may be considered among the main non-point sources of anti-infectives in the aquatic environment.

## Fate and effects

Once in the aquatic environment, anti-infectives are affected by different abiotic and biotic processes influencing their bioavailability and their subsequent impact on aquatic biota. The relative importance of these processes on the fate of anti-infectives is dictated by their physicochemical properties as well as by the conditions of the medium in which they are present (Peschka et al. 2007). Biodegradation and nonbiological processes such as sorption, photolysis, and hydrolysis may reduce anti-infective loads in the environment and result in partial degradation or mineralization of these compounds (Alexy 2004; Halling-Sørensen et al. 1998). Compounds having a lower susceptibility to these processes may be persistent, in which case their environmental concentrations are reduced mainly through dilution in the aquatic environment. However, anti-infectives do not need to be very persistent in the environment to be able to have an effect. Contrary to other organic pollutants such as pesticides (Comoretto and Chiron 2005), anti-infectives are constantly released in the environment; therefore, substances degrading in a few days can be considered persistent with respect to natural waters at the point of discharge where releases are continuous (Sedlak et al. 2004).

## Anti-infective resistance

To date, the main interest for the study of anti-infectives in the environment has focused on their potential role on the spread of anti-infective resistance, as these substances are specifically designed and used with the purpose of inhibiting microbes. Anti-infective resistance is defined as the ability of a population of microorganism cells to neutralize the toxic effect of an anti-infective (Suling and O’Leary 1975). When a bacterium develops resistance to a particular anti-infective mode of action, either by the acquisition of genes via mobile agents (e.g., plasmids and transposons) or by means of mutations, it gains an evolutionary advantage over its nonresistant congeners when the host is under anti-infective treatment (Levy and Marshall 2004). However, this advantage ends once therapy on the patient is over, because the recolonization of the medium by nonresistant bacteria will not be impeded and, as a consequence, resistant strains become diluted (Levy and Marshall 2004).

So is it possible that anti-infective occurrence in environmental waters promotes resistance? Several authors have argued that if concentrations are higher than the minimum inhibitory concentrations (MICs) of some pathogenic bacteria, a selective pressure would be exerted and, as a result, the dissemination of anti-infective resistance could be enhanced (Kümmerer and Henninger 2003). It has also been proven that even subinhibitory concentrations (e.g., one-fourth of the MIC) of several anti-infectives are able to have an effect [e.g., as shown for the modulation of the expression of toxin-encoding genes in *Staphylococcus aureus* (Ohlsen et al. 1998)]. Transfer of genetic elements among bacteria has been observed under natural conditions in WWTPs (Marcinek et al. 1998), and selection of resistant bacteria has been documented in sewers receiving wastewaters from pharmaceutical plants (Guardabassi et al. 1998). Agricultural activities may also contribute to the transfer of resistance genes from wastewater bacteria to indigenous soil microbiota (Chee-Sanford et al. 2001). However, the extent of the impact of the occurrence of anti-infectives in the aquatic environment on the dissemination of resistance in bacteria is still a subject of debate (Ohlsen et al. 2003; Summers 2002), and present knowledge indicates that its impact is still questionable (Kümmerer 2004).

## Ecosystem health

Anti-infectives are biologically active substances; therefore, they pose a potential threat to aquatic biota. Recently, numerous studies have reported the acute and chronic toxic effects of anti-infectives on nontarget organisms such as diatoms (Wilson et al. 2003), algae (Ferrari et al. 2004; Halling-Sørensen 2000), crustaceans (Ferrari et al. 2004; Flaherty and Dodson 2005; Isidori et al. 2005), rotifers (Ferrari et al. 2004; Isidori et al. 2005), cnidarians (Quinn et al. 2008), and amphipods (Maul et al. 2006). These reports indicate that effective concentrations of most studied aquatic species are 2–5 orders of magnitude higher than those generally found in contaminated natural waters. Nevertheless, sensitive species such as diatoms, green algae, cyanobacteria, and some pathogenic bacteria (Al-Ahmad et al. 1999; Kümmerer et al. 2000; Wilson et al. 2003) are affected by concentrations < 2,000 ng/L. We must also consider that most of these studies target effects using a single species and single compounds. Surface waters near WWTP outfalls contain a myriad of organic and inorganic compounds that can interact as toxic mixtures. In comparison to short-term exposures in the laboratory, chronic exposures are likely to result in observable effects at lower thresholds. Other changes in the environment, indirect effects or more subtle effects that may affect species such as food selection behavior (Daughton and Ternes 1999; Hahn and Schulz 2007), or the fate of other organic pollutants such as pesticides should also be contemplated (Boxall et al. 2003).

## Methods

### Selection and classification of literature data

Because an enormous amount of data has been published over the last 24 years on the occurrence of anti-infectives in the environment, we decided to set the following criteria to select and assure the quality of the compiled values. Only data reported in peer-reviewed journals written in the English language were considered for compilation. Only articles indicating or citing the limit of quantification (LOQ) of their determination method were selected. Therefore, only values higher than or equal to the reported LOQ were considered. When the authors reported only the limit of detection (LOD), the LOQ was evaluated by multiplying the LOD by a conversion factor *x* according to the definition of the LOD used by the authors. For example, when the authors defined the LOD as the concentration giving a signal-to-noise ratio (S/N) of 3, the LOQ was calculated by multiplying the LOD by a factor *x* = 3.3, as the LOQ is equivalent to S/N = 10. In addition, when the same substance was analyzed by more than one method in the same study, the concentration reported using the most sensitive method (lower LOQ) was used. When the authors did not report any concentrations in real samples, their LOQ values were selected for compilation. Only concentrations in which the type (e.g., raw sewage, surface water) and the country of provenance of the sample were clearly indicated were used. Only data reported as numeric values were used. Data reported in figures were not considered because of the uncertainty of their interpretation. Only natural concentrations were reported; values in natural waters with experimental amendment of manure or sediments were not considered. Urban effluents were classified in three categories according to the treatment applied: primary (physical or mechanical), secondary (biological), and tertiary (advanced, such as disinfection by chlorination or ultraviolet radiation) (vanLoon and Duffy 2000). Waters found in agricultural matrices such as runoff, lagoons, and field streams were classified as wastewaters. Ecotoxicologic data such as lowest observed effective concentration (LOEC) and 50% effective concentration (EC_50_) of sulfamethoxazole and ofloxacin for several types of bacteria and aquatic species were gathered from the peer-reviewed literature. We included as many different species as possible to illustrate the distribution of effective concentrations on aquatic biota of these two anti-infectives.

### Statistical analysis

The distribution of anti-infective concentrations in the different matrices are described by their arithmetic mean, median, 75th and 95th percentiles, and maximal concentrations. We mined the data looking for expected trends or for possible relations with factors such as the geographic region, anti-infective class, and the treatment process, if any. The matrix of selected data cannot be processed entirely through statistical analyses because of the heterogeneity of the studied compounds between matrices and countries. However, focusing on urban wastewater and on three data-rich classes (macrolides, quinolones, sulfonamides) and one compound (trimethoprim), we compared their concentrations in raw and treated wastewaters from three different regions (East Asia, Europe, and North America). An analysis of variance with the general linear model procedures of SPSS (version 16.0; SPSS Inc., Chicago, IL, USA) was used for that purpose. Density histograms and normal density functions of sulfamethoxazole, ofloxacin, and their LOEC and EC_50_ were calculated by Systat (version 11.0; Systat Software Inc., Chicago, IL, USA).

## Results and Discussion

### Occurrence of anti-infectives in the environment

A bibliographic search of the scientific literature found 159 articles published between 1984 and mid-2008 reporting analyses of anti-infectives in wastewaters, surface waters, or drinking waters. The attention that the occurrence of anti-infectives in the aquatic environment has generated in recent years is reflected by the number of papers published each 5-year period since 1984: 2 (1984–1988), 0 (1989–1993), 6 (1994–1998), 27 (1999–2003), and 124 (2004–2008). Most of the studies we found reported concentrations of these compounds in environmental waters coming from countries defined as having high incomes (World Bank 2008); in fact, only 28 articles of 159 originally found (18%) analyzed waters from low- to middle-income countries.

Applying the selection criteria enumerated in the “Methods,” we eliminated 16 articles. A database was built containing > 2,200 values of concentrations and 2,500 LOQ values of anti-infectives in drinking, natural, and waste-waters reported in the 143 selected publications. A summary of the detection instruments showed than 75% of the reported values come from methods using tandem mass spectrometry, 14% from single mass spectrometry, and 11% from molecular spectroscopy (fluorescence or ultraviolet). By compiling the data obtained from different analytical methods and sources, we tried to offset a bias in our results caused by the publications reporting more values. In total, from the 126 different substances (parent compounds, degradation products, or metabolites) for which at least one method of determination exists, only 68 different parent compounds and 10 degradation products or metabolites have been quantified so far in environmental waters. Table 1 shows occurrence data sets organized by anti-infective class and matrix. For more detailed information, see Supplemental Material, Table 1 (available online at http://www.ehponline.org/members/2009/11776/suppl.pdf).

Several classes of anti-infectives have been less frequently reported in spite of the numerous studies that have tried to determine them. One example is the β-lactams, for which only 12 of 24 different compounds have been determined so far in environmental waters. As mentioned earlier, this is due to their high reactivity in aquatic media. Also, among the most important classes of anti-infectives that have not yet been determined are the quinoxaline dioxides (carbadox, olaquindox), which at least 13 different studies have been unable to quantitate.

The study of metabolites is important for the accurate determination of anti-infectives, as some conjugated metabolites, such as glucuronide, sulfate, and *N*-acetyl, can be deconjugated in wastewaters by bacterial enzymes, thus increasing the concentration of the parent drug (Jones et al. 2005). Few studies have looked into this problem, and only *N*^4^-acetylsulfamethoxazole, a metabolite of the sulfonamide sulfamethoxazole, has been quantitated to date (Ashton et al. 2004; Göbel et al. 2004, 2005, 2007; Hilton and Thomas 2003). As for degradation products, their occurrence is less significant in the environment if they are not as biologically active as the parent drug. This is the case of anhydro-erythromycin (erythromycin-H_2_O), the degradation product of the macrolide erythromycin, which has been widely quantitated in the literature. Other degradation products such as those of the β-lactams or the tetracyclines have been only sparsely documented (Li et al. 2008b; Mackie et al. 2006).

An important aspect of our study that we must emphasize is that the selected occurrence data are not representative of the water matrices or the global state of water contamination with respect to anti-infectives. Published values are biased, because analyzed samples are often collected in sites where contamination is suspected. In addition, information on frequency of detection is not always available, which also overestimates the occurrence of anti-infectives. Therefore, it must be kept in mind that throughout our study, our results and conclusions apply mostly to contaminated waters.

### Drinking water

Occurrence of anti-infectives in drinking water is the least reported so far. Only about 2% (3 of 143 selected papers) indicate quantitative values in drinking water, even though more than eight different studies have tried to measure them. This can be explained by the low limits of quantification necessary to achieve their determination in drinking water, which often must be < 1 ng/L. Anti-infective concentrations in contaminated tap water range from 0.3 to 5 ng/L, with a median concentration of 2 ng/L. We found only one study that attempted to measure the concentration of degradation products or metabolites. Anti-infectives reach drinking water, albeit in very low amounts, because they are able to persist in natural water sources and resist purification processes in drinking water treatment plants (DWTPs). However, anti-infectives seem to be more affected by purification processes than other, more frequently reported organic wastewater contaminants (OWCs). In a study on the fate of 106 OWCs (including 25 anti-infectives) in a conventional DWTP using several physicochemical processes in sequence, from the 42 OWCs detected above their reporting limit in stream and raw water samples, only five were anti-infectives. In finished waters, only 17 OWCs were detected, and none of them were anti-infectives (Stackelberg et al. 2004). A study on the effectiveness of several treatment processes used in DWTPs showed that activated carbon sorption, reverse osmosis, and oxidation (chlorination or ozonation) were among the most efficient treatments to remove anti-infectives from source water (Adams et al. 2002).

### Natural waters

Occurrence of anti-infectives has been well documented in both groundwater and surface waters (44% of selected articles). Rivers, creeks, lakes, estuaries, basins, sea waters, and wells have been reported to be contaminated by several of these compounds. Values found in the literature show up to eight orders of magnitude of variation, and concentrations often decrease as the distance from the source (WWTP outfalls, landfills, etc.) increases. The cause of the attenuation of anti-infectives in surface waters cannot be easily interpreted, because different attenuation mechanisms may operate simultaneously (Sedlak et al. 2004). Published data in the literature do not indicate the present state of the global anti- infective contamination of surface waters, and this may appear overestimated by the choice of sampling points, often near WWTP outfalls or agricultural areas. For example, a study of 139 streams in the United States showed that only 2 of the 23 targeted anti-infectives were detected in > 20% of the samples collected in zones susceptible of contamination (Kolpin et al. 2002). These observations were later confirmed by Focazio et al. (2008), who showed that only 6 anti-infectives from the 25 initially targeted were found in < 35% of the 74 untreated drinking water sources across the United States.

### Surface waters

As the receptors of most WWTP final effluents, outfalls in streams and the nearby downstream zones act as collectors of all the substances that were not removed by the treatment process. Our database showed that 52 of 143 papers (36%) report concentrations of anti-infectives in surface waters. Median concentration of these drugs in contaminated surface waters was 30 ng/L, and values varied between 0.07 and 712,000 ng/L. As for metabolites and degradation products, their median concentration was 548 ng/L, and reported amounts ranged from 2–10,540,000 ng/L. Detection of anti-infectives in surface waters upstream of WWTPs outfalls indicate the persistence and mobility of these compounds, which may be discharged by point or nonpoint sources. The importance of these sources on the anti-infective occurrence in downstream surface water depends clearly on the season and hydrology of the region. Kolpin et al. (2004) demonstrated that stream flow conditions significantly affect occurrence of organic wastewater contaminants, as higher flows increase the dilution factor of WWTP effluents. Also, the contribution of WWTP effluents to the total flow of rivers is determinant. Rivers in which the majority of the flow is composed of WWTP effluent will have a lower diluting power (some rivers are composed of up to 75–80% of WWTP discharges), and anti-infective concentrations downstream will be approximately constant, depending on other attenuation processes such as photolysis or sorption (Bendz et al. 2005; Hirsch et al. 1999).

### Groundwaters

Groundwaters are affected by a variety of sources, with landfills, septic systems, and agricultural fields representing the most significant potential sources of anti-infective contamination. A recent national reconnaissance study of 65 OWCs in groundwaters in the United States (Barnes et al. 2008) detected only 3 anti-infectives of the 21 targeted. Sulfamethoxazole was the most frequently detected (23.4%), and its maximum concentration was 1,110 ng/L. However, the detected anti-infectives represented < 5% of the total OWCs concentration. According to our database, 10% (13 of 143 sampled articles) reported concentrations of anti-infectives in groundwaters in the range of 0.2–1,400 ng/L, and a median concentration of 71 ng/L. Only one paper, a study on the occurrence of sulfonamides in ground-waters near a pharmaceutical waste landfill in Denmark (Holm et al. 1995), reported higher amounts, with a median concentration of 190,000 ng/L and a maximum concentration as high as 1,600,000 ng/L. Landfills containing WWTP biosolids or discarded anti-infectives contaminate groundwaters because leachate plumes may reach nearby aquifers. In addition, as opposed to surface waters, anaerobic or suboxic conditions are often observed in groundwaters and may prevent or slow down the degradation rates of some anti-infectives (Verstraeten et al. 2005). Studies on the disposal of pharmaceuticals in the United States and the United Kingdom (Bound and Voulvoulis 2005; Kuspis and Krenzelok 1996) showed that a significant proportion of people (54% in the United States, 71% in the United Kingdom) disposed of unused medication in the trash. Hence, the role of landfills in the contamination of groundwaters should be reassessed, as disposal of anti-infectives is usually considered only a minor source of contamination (Boxall 2004).

### Wastewaters

Wastewaters produced by urban centers as well as by agricultural activities were the most studied matrices in the selected literature (62%). Reported concentrations in this matrix are obviously the highest, but they vary by up to 10 orders of magnitude. This huge variability is mainly a consequence of the diverse origin of the waste-waters, which may come from industries, hospitals, municipal WWTPs, farm lagoons, field runoff, and so on. Also, anti-infective concentrations are affected by the different treatment process applied to wastewaters, which in some cases are nonexistent (as is the case of direct discharges of urban or agricultural origin) and in others very advanced, such as tertiary wastewater treatment systems that include reverse osmosis and micro- and nanofiltration as well as ozonation.

### Industrial sewage

Manufacture of anti-infectives often generates highly contaminated sewage. For example, biosynthetic fabrication of tetracyclines produces wastes having high chemical oxygen demand (COD) loads, and treatment of these waters is both difficult and expensive (Li et al. 2004). Consequently, anti-infective concentrations in these manufacturing wastes are worryingly high, as is the case of wastewaters from oxytetracycline production facilities reaching values as high as 920,000,000 ng/L (Li et al. 2008a), which are several times higher than the EC_50_ for some aquatic species such as *Microcystis aeruginosa* (EC_50_ = 20,700 ng/L) or *Rhodomonas salina* (EC_50_ = 160,000 ng/L) (Holten-Lützhøft et al. 1999). Other compounds such as the quinolones have also been reported in effluents from drug manufacturers (Larsson et al. 2007) with concentrations of ciprofloxacin up to 30,000,000 ng/L, which are well above EC_50_ values for several aquatic species as well (Larsson et al. 2007). The mixing of industrial wastes with human sewage creates further concerns because it generates an ideal environment for spreading anti-infective resistance in bacteria (Larsson et al. 2007). The β-lactams and their metabolites have been also reported in manufacturing plant effluents, with a concentration of benzylpenicillin (153,000 ng/L) comparable to published MICs (Li et al. 2008b).

According to our bibliographic research, about 5% (7 of 143 of the sampled articles) report the presence of anti-infectives in industrial wastewaters and concentrations range from 4,900–920,000,000 ng/L, with a median concentration of 300,000 ng/L. The extent of the contribution coming from manufacturing plants to the overall occurrence of anti-infectives in the environment cannot be evaluated at present, because published data are still scarce. (These reports were limited to only three countries: China, India, and Croatia). Industrial discharges in the environment in high-income countries like the United States are controlled by current good manufacturing practices and emissions regulations (Velagaleti et al. 2002); therefore, the impact of drug manufacturers should be limited only to countries with more flexible legislation (or lacking the resources to enforce them). In 1999, low- to middle-income countries accounted for only 7.1% (by value) of the world pharmaceutical production; never-the less, at least 10 low- to middle-income countries produce active ingredients, with China and India leading this group (World Health Organization 2004). In countries with less strict regulations, anti-infective production facilities may be among the most important sources of these substances in their nearby aquatic environment (Larsson et al. 2007).

### Hospital sewage

Hospitals are considered one of the most important sources of anti-infectives in the aquatic environment (Gómez et al. 2006). However, < 8% of the selected papers (12 of 143) report the occurrence of these compounds in hospital sewage. Concentrations of anti-infectives in contaminated hospital waters range from 10–124,500 ng/L, with a median value of 2,100 ng/L. The maximum concentration of ciprofloxacin found in hospital effluents (124,500 ng/L) (Hartmann et al. 1988) is considerably higher than the lowest effect concentration of ciprofloxacin for genotoxicty (LOEC = 200 ng/L) or the EC_50_ of some pathogens (EC_50_ = 2000 ng/L) (Kümmerer et al. 2000). Quinolones, especially ciprofloxacin, were the main cause of the DNA-damaging effects detected in wastewater samples from hospitals (Hartmann et al. 1988). Additionally, compared with antineoplastic drugs, quinolones have a greater potential to cause DNA damage (Hartmann 1999). These findings support the concern for the potential impact of anti-infective residues on the spread of bacterial resistance, although according to other studies, the concentrations of anti-infectives found in hospital wastewaters are below the concentrations known to promote resistance (Jarnheimer et al. 2004; Ohlsen et al. 2003).

Although detection frequencies and concentrations are generally higher in hospital sewage than in municipal wastewaters, it has also been reported that anti-infective concentrations in hospital sewage are similar or lower than the concentrations found in municipal WWTP influents (Karthikeyan and Meyer 2006) or retirement homes (Brown et al. 2006). Therefore, the impact of hospitals may depend on the communities, the season, and water use, but results tend to indicate that wherever large groups of individuals under medication cohabit in the same location, significant concentrations of anti-infectives will be found in sewage.

### Agricultural and aquacultural waste-waters

Papers reporting the presence of anti-infectives in agricultural waters coming from hog, fish, and shrimp breeding are among the first reports published in the anti-infectives in the environment literature (Migliore et al. 1996; Smith et al. 1994). These studies reflect early concerns on the intense use of these compounds in farming and their fate. About 7% of the selected articles (10 of 143) report values in agricultural wastewaters. Concentrations of anti-infectives in lagoons or aquaculture ponds range between 1 and 13,000,000 ng/L, with a median concentration of 22,930 ng/L. Residues of these drugs in aquaculture waters may reach high values because they are used as feed additives; they may leach from the food pellets and are also excreted by the animals. Sediments may also accumulate anti-infectives, which may be released later in the water. Estimates indicate that 70–80% of drugs used as feed in aquaculture may reach the environment (Holten-Lützhøft et al. 1999; Migliore et al. 1996).

Occurrence of anti-infectives in field tiles, field streams, and runoff show much lower concentrations (2–4,000 ng/L). Differences in concentrations for anti-infectives in these matrices may be due to several factors such as the dose used, resistance to biodegradation, and mobility, as well as soil characteristics. Because the mobility of anti-infectives is affected by their affinity to particulate matter, their occurrence in overland flow seems to be more important for rather hydrophilic compounds such as the sulfonamides or trimethoprim than more hydrophobic or binding-capable compounds such as the tetracyclines or the macrolides (Kay et al. 2004). However, compounds having high sorption coefficients are still able to be transported and reach environmental waters (Kay et al. 2004). Sorption of more hydrophilic compounds (e.g., sulfonamides) onto soils may increase with time, thus reducing their release into environmental waters (Stoob et al. 2007). The influence of other transport process such as binding to dissolved organic matter remains unclear (Lee et al. 2007).

### Urban wastewaters

Because of the preponderant role of WWTPs on the anti-infective contamination of surface waters, urban wastewaters have been extensively studied in the past 10 years. In fact, 51% of the sampled papers reported concentrations in urban wastewaters. In our database, occurrence of anti-infectives in urban raw sewage range from 3–10,570 ng/L, with a median concentration of 300 ng/L. In treated effluents, concentrations vary between 1 and 29,000 ng/L and a median of 136 ng/L. Analyses in raw sewage and WWTP effluents applying different types of processes have demonstrated the failure of the commonly used wastewater treatment technologies to completely remove anti-infectives present in wastewaters.

As our bibliographic research of urban wastewaters provides enough data for three classes of substances [macrolides (including anhydro-erythromycin, the degradation product of erythromycin), quinolones, and sulfonamides] and one compound (trimethoprim), four levels of treatment (none, primary, secondary, and tertiary) in three geographic areas: East Asia (China, Japan, and South Korea), Europe (Austria, Croatia, Denmark, France, Finland, Germany, Greece, Italy, Norway, Sweden, Spain, Switzerland, and the United Kingdom), and North America (Canada, Mexico, and the United States), it was possible to investigate the influence of these factors on anti-infective concentrations. Analysis of variance with Dunnet’s T3 post-hoc tests showed significantly higher (*p* < 0.05) concentrations in raw wastewaters compared with primary, secondary, and tertiary effluents. Analysis of variance also showed that the concentrations measured in North America and East Asia were significantly higher than those in Europe. Furthermore, resulting concentrations of sulfonamides and trimethoprim are significantly higher than those of macrolides and quinolones. These results confirm previous observations about the removal of anti-infectives in WWTPs (Batt et al. 2007; Göbel et al. 2005; Gulkowska et al. 2008; Ternes et al. 2004). Removal efficiency is dependent on many factors, the most important being the type of treatment (e.g., primary, secondary), the WWTP design (hydraulic and solid retention times, sludge age, etc.), and the physicochemical properties of each compound (e.g., pKa and log D_ow_).

#### Ecologic significance of ambient concentrations of anti-infectives

##### The case of sulfamethoxazole and ofloxacin

**Figure 1:**
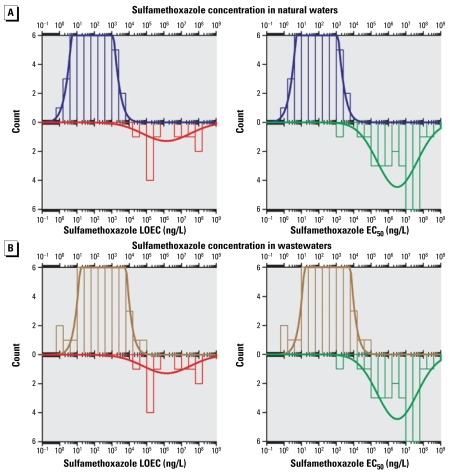
Density histogram (bars) and density function (line) of sulfamethoxazole occurence in natural waters (*A*) and wastewaters (*B*) compared with density histogram and density function of LOEC (left) and EC_50_ (right) values for several aquatic species exposed to sulfamethoxazole.

Sulfamethoxazole and ofloxacin were chosen for further insight into the significance of the occurrence data reported in the literature from an ecotoxicologic standpoint. Figure 1 shows the histogram and the normal density function of sulfamethoxazole for natural and waste-waters and the LOEC and EC_50_ for different species [see Supplemental Material, Table 2 (available online at http://www.ehponline.org/members/2009/11776/suppl.pdf)]. The distribution of sulfamethoxazole concentration in natural waters clusters around 10^2^ ng/L, and for wastewaters this value is about an order of magnitude higher. This difference coincides well with fate data, showing a rather weak affinity of sulfamethoxazole for solids, which enhances its transport in the aqueous phase. Ecotoxicologic values show a much wider distribution, with a density function maximum around 10^6^ ng/L for LOEC and 10^7^ ng/L for EC_50_. In some cases, these density curves lie over common ranges, and we interpreted this overlapping as an increased risk for aquatic species. We observed that < 1% of LOEC values and < 0.1% of the EC_50_ values were lower than the highest 10% of the concentrations of sulfamethoxazole in natural water. When looking at the density curves of effective concentrations versus occurrence of sulfamethoxazole in wastewaters, we can see that overlapping between them is slightly more important. About 3% of LOEC and < 1% of EC_50_ values were lower than the highest 10% of the concentrations of sulfamethoxazole in wastewaters. The presence of this overlapping region suggests that the observed concentrations of sulfamethoxazole in natural waters are not high enough to affect most studied aquatic species but that concentrations in wastewaters could have in impact on the most sensitive species such as bacteria.

**Figure 2:**
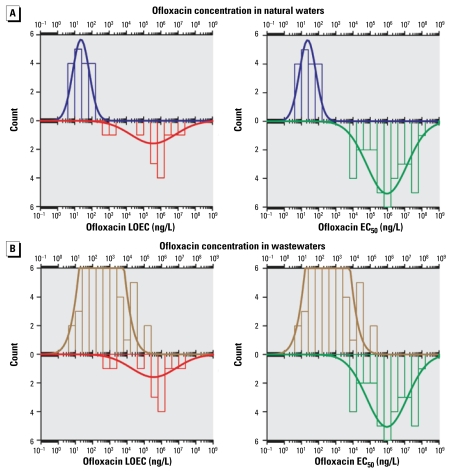
Density histogram (bars) and density function (line) of ofloxacin occurrence in natural waters (*A*) and wastewaters (*B*) compared with density histogram and density function of LOEC (left) and EC_50_ (right) values for several aqualtic species exposed to ofloxacin.

In the case of ofloxacin (Figure 2), the distribution of its concentration shows that occurrence of this quinolone in natural waters centers around 10^2^ ng/L, whereas in waste-waters this value is between 1 and 2 orders of magnitude higher. Compared with sulfamethoxazole, this difference is more pronounced, which is not surprising given the higher affinity of ofloxacin for solids and its subsequent better elimination by waste-water treatments. As with sulfamethoxazole, ecotoxicologic values [see Supplemental Material, Table 3 (available online at http://www.ehponline.org/members/2009/11776/suppl.pdf)] show a wide distribution, having a maximum close to 10^5^ ng/L for LOEC and 10^6^ ng/L for EC_50_. Less than 1% of LOEC values and < 0.1% of the EC_50_ density curves were lower than the highest 10% of concentrations of ofloxacin in natural water. Also, the density curve overlapping of wastewaters and effective concentrations is much more important than in the case of sulfamethoxazole, with around 8% of LOEC values and < 2% of the EC_50_ values being lower than the highest 10% of concentrations of ofloxacin in wastewaters. Therefore, ofloxacin seems to present a higher ecotoxicologic risk than sulfamethoxazole, and detrimental effects on wastewater bacteria are more likely to occur than in aquatic biota.

Thus, what is the ecotoxicologic significance of the occurrence of anti-infectives in environmental waters? Assuming that our sampled data are representative of ambient anti-infective concentrations in most contaminated environmental waters, it can be argued that even a weak overlapping between concentration values corresponding to environmental waters and ecotoxicologic data could have detrimental effects on the most sensitive species such as bacteria or algae. In the case of surface waters, because anti-infectives are constantly being released into the environment, microbiota are constantly exposed to these compounds. These harmful effects should be more important in small streams affected by urban or agricultural discharges, because of their reduced dilution capacity. With regard to wastewaters, even if our results show that high concentrations (> 10,000 ng/L) of anti-infectives in these waters are more the exception than the rule, the existence of a few locations where these concentrations can be reached are enough to contribute to the global spreading of anti-infective resistance (Okeke and Edelman 2001). Given that large populations of bacteria are being exposed to a selective pressure, environmental waters and especially wastewaters become ideal settings for the assembly and exchange of mobile genetic agents encoding for resistance in bacteria (O’Brien 2002).

#### Additional factors to be considered

##### Mixture effects

Mixture effects are expected in environmental waters because many other organic and inorganic contaminants are discharged in conjunction with different anti-infectives. According to a recent study using predicted environmental concentrations, when strong synergistic effects are present between anti-infectives and other pharmaceuticals occurring in wastewater, an impact on resistance in bacteria is possible but not in fungi (Kostich and Lazorchak 2008). Certain substances that may be present in environmental waters have a synergistic effect on some anti-infectives. For example the MIC of ampicillin for *Pseudomonas aureginosa* is 1,500 mg/L, but in combination with 500 mg/L EDTA, the MIC was reduced to 22 mg/L (Lambert et al. 2004). Surfactants have also been reported as potentiators (agents capable of enhancing the activity of a substance) of chlortetracycline and benzylpenicillin in certain bacterial strains (Suling and O’Leary 1975). In contrast, antagonistic interactions between anti-infectives and other pharmaceuticals on *Escherichia coli* and human ovarian carcinoma cells have been observed *in vitro* with environmentally realistic concentrations (Pomati et al. 2008). Thus, more information is necessary to better predict the effect of chronic exposure to complex mixtures such as surface waters near WWTP outfalls.

### Occurrence of anti-infectives in low- to middle-income countries

Our bibliographic research showed an important gap in the present knowledge of anti-infective contamination on a global scale with regard to low- to middle-income countries. From the total 159 articles identified, 84% analyzed environmental waters in high-income countries; the remaining 16% are from four Asian countries (China, India, Malaysia, and Vietnam), two European countries (Croatia and Poland), and one Latin American country (Mexico). We did not find any data in the peer-reviewed literature concerning the environmental occurrence of anti-infectives in Africa or in other Asian or Latin American countries. In 1999, low- and middle-income countries consumed < 10% (by value) of the world’s medicines (World Health Organization 2004). Nevertheless, the high rates of over-the-counter self-medication (Kamat and Nichter 1998; Kunin 1993) and wide availability of inexpensive anti-infectives combined with a lower access to public sewage networks could result in environmental waters containing significantly higher amounts of anti-infectives in these countries than in high-income countries. Therefore, more research focusing on the occurrence of these substances in low- and middle-income countries is necessary to properly evaluate the state and impact of global contamination of waters. In addition, discharge from anti-infective manufacturing in low- to middle-income countries needs to be studied, as their input could actually be the most important point source in the local environment of some regions (Larsson et al. 2007). If anti-infective occurrence in environmental waters does promote resistance in bacteria, the contamination caused by anti-infectives should be approached from a global perspective, as people and products affected by these waters can contribute to the spread of anti-infective resistance to other parts of the world (O’Brien 2002; Okeke and Edelman 2001).

## Conclusion

Anti-infectives, the miracle drugs of the 20th century, have become environmental contaminants of emerging concern in the 21st century. Research has shown that these compounds are persistent and mobile enough to be transported from landfills, agricultural fields, and urban centers to natural waters. To gain a better insight of the global contamination caused by anti-infectives in environmental waters, we created a database with more than 2,200 concentration values of 68 parent drugs and 10 metabolites or degradation products reported in 143 peer-reviewed papers. Statistical analysis of concentrations of three classes of anti-infectives (macrolides, quinolones, and sulfonamides) and trimethoprim in urban wastewaters in three geographic areas (East Asia, Europe, and North America) confirmed significantly higher concentrations in raw wastewaters compared with treated wastewaters. Also, concentrations measured in Europe were significantly lower than those in North America and East Asia. Furthermore, resulting concentrations of sulfonamides and trimethoprim are significantly higher than those of macrolides and quinolones. These results confirm previous observations about the factors influencing the removal of anti-infectives. Comparison between sulfamethoxazole and ofloxacin occurrence in natural and wastewater and their effective concentrations in aquatic biota showed that there is a weak overlapping of the distribution curves and that only highly contaminated waters could affect the most sensitive species. However, potential effects on aquatic microbiota cannot be ruled out for the following reasons:

- Effects of chronic exposure of sensitive organisms such as bacteria or algae to sub-inhibitory concentrations of anti-infectives over long periods of time are still unknown.
- Even if highly contaminated wastewaters are rather rare with respect to anti-infectives, heavily impacted industrial or agricultural wastewaters could become a nonnegligible environmental reservoir of anti-infective resistant bacteria, given that they have all the necessary elements of an ideal setting for the assembly and exchange of mobile genetic agents encoding for resistance.
- Current knowledge on the global occurrence of anti-infectives in environmental waters is far from complete. More research is necessary, especially for low- to middle-income countries, which may be more impacted by anti-infective contamination than high-income countries because of less extended public sewage infrastructures, higher rates of self-prescription, and often less-strict industrial emissions legislations.
- The effects of cumulative and synergistic effects of anti-infectives in complex mixtures such as wastewaters are yet to be unraveled.

The current tendency toward larger and more densely populated production facilities, such as concentrated animal feeding operations, suggests that occurrence of anti-infectives in agricultural wastewaters may increase in the near future (Lee et al. 2007). Also, water-saving policies in urban settings would result in a reduction of wastewater volumes and consequently, in the increase of anti-infective levels because of lower dilution (Kümmerer 2004). Many measures to avoid the presence of pharmaceuticals in the environment have been proposed so far. Two main approaches in urban settings can be distinguished: source control and improvement of wastewater technologies. Source control solutions look for the reduction of pharmaceutical inputs before they reach public sewer systems, at the consumer level (e.g., environmental labeling to inform patients and physicians) or at the waste management level [e.g., urine separation (Larsen et al. 2004)], as well as pretreatment of hospital sewage (Ternes et al. 2004). Improvement of sewage treatment processes to increase removal efficacy of WWTPs includes optimization of current technology and the implementation of more advanced treatment techniques such as ozonation, advanced oxidation processes, membrane filtration, and activated carbon (Ternes and Joss 2006; Ternes et al. 2003). With regard to anti-infectives of agricultural origin, better farming practices have been proposed such as erosion control to reduce runoff (Davis et al. 2006), increasing the maturation time of manure before application to enhance degradation (De Liguoro et al. 2003), and the use of filters to reduce discharges by aquaculture operations (Smith et al. 1994). All of these measures should contribute to the reduction of urban and agricultural inputs of anti-infectives in the aquatic environment.

## Figures and Tables

**Table 1:** Occurrence in nanograms per liter of anti-infectives in contaminated wastewaters and natural and drinking waters organized by class and matrix.

Anti-infective class	No. > LOQ^a^	No. papers^b^	Mean	Median	75%	95%	Max	LOQ_low_^c^	LOQ_high_^d^
Wastewaters									

As parent compounds									

Azoles	17	6	5,987	26	50	61,920	90,200	5	112
β-Lactams	42	15	4,633	300	1,200	5,360	153,000	1	100,000
Quinolaxine-dioxide	0	5						5	100
Lincosamides	33	10	18,715	60	2,125	190,500	240,000	0.3	100
Macrolides	217	43	352	110	271	1,000	27,000	0.2	1,155
Poliether ionophores	13	2	29	11	26	167	190	1	3
Quinolones	420	51	152,247	205	570	41,922	31,000,000	1	20,600
Sulfonamides	289	57	11,972	330	800	31,000	1,158,680	1	300,000
Tetracyclines	161	32	11,642,200	530	7,250	6,095,000	920,000,000	1	700,000
Trimethoprim^e^	210	52	1,351	270	795	5,000	55,200	1	150,000
Other	17	11	750	39	1,115	4,101	5,000	3	667

As metabolites/degradation products									

β-Lactams	10	1	55,794,600	7,630,000	44,500,000	389,000,000	389,000,000	410	1,360
Macrolides	52	16	2,848	450	1,414	5,650	83,000	1	500
Sulfonamides	13	5	779	570	1,200	2,230	2,235	20	212
Tetracyclines	6	1	5,092,167	1,445,000	9,420,000	18,100,000	18,100,000	600	1,300

Natural waters									

As parent compounds									

Azoles	39	11	18	18	23	44	58	1	370
β-Lactams	6	16	73	11	48	350	350	2	24,000
Quinolaxine-dioxide	0	9						35	1,400
Lincosamides	46	21	147	18	100	1,020	1,400	0.04	198
Macrolides	128	38	58	11	46	197	1,022	0.02	1,155
Poliether ionophores	4	4	312	35	606	1,172	1,172	0.1	380
Quinolones	78	31	199	27	108	640	5,600	0.3	7,000
Sulfonamides	234	60	66,531	120	700	472,000	1,600,000	0.2	33,000
Tetracyclines	47	33	97,369	192	658	623,550	712,000	0.07	1,650
Trimethoprim	90	24	94	18	42	510	3,000	0.2	4,000
Other	5	21	136	127	207	266	266	2	2,000

As metabolites/degradation products									

β-Lactams	4	1	4,719,500	4,037,500	8,840,000	10,540,000	10,540,000	410	1,360
Macrolides	51	24	184	40	146	1,186	1,700	0.3	250
Sulfonamides	6	6	86	14	239	240	240	2	50
Tetracyclines	27	25	12,367	11,100	14,750	32,840	34,200	10	1,300

Drinking waters									

As parent compounds									

Azoles	0	1						370	370
β-Lactams	0	0						NA	NA
Quinolaxine-dioxide	0	1						100	100
Lincosamides	0	2						0.07	5
Macrolides	4	2	3	3	5	5	5	0.07	220
Poliether ionophores	0	0						NA	NA
Quinolones	5	2	2	2	3	4	4	0.3	10
Sulfonamides	2	5	0.4	0.4	0.5	0.5	0.5	0.2	1,155
Tetracyclines	0	1						3	12
Trimethoprim	0	4						0.5	250
Other	0	1						5	5

As metabolites/degradation products									

β-Lactams	0	0						NA	NA
Macrolides	0	1						10	10
Sulfonamides	0	0						NA	NA
Tetracyclines	0	0						NA	NA

NA, not available.

a Number of values reported as being > LOQ for each class.

b Number of papers reporting analysis in each matrix for each class.

c Lowest LOQ reported.

d Highest LOQ reported.

e Dihydro-folate reductase inhibitor.
